# Management of the vaccination campaign in a population of frail older outpatients affected by cognitive or endocrinological conditions: a pilot study in Italy

**DOI:** 10.1007/s40520-024-02824-5

**Published:** 2024-08-30

**Authors:** Nicola Veronese, Francesco Saverio Ragusa, Pascal Roberto Titone, Laura Vernuccio, Giuseppina Catanese, Maria Angela Randazzo, Mario Palermo, Giovanna Di Bella, Pasquale Mansueto, Ligia J. Dominguez, Mario Barbagallo

**Affiliations:** 1 Department of Health Promotion, Mother and Child Care, Internal Medicine and Medical Specialties, Geriatrics Section, University of Palermo, Palermo, Italy; 2 Prevention and Epidemiology Unit, Palermo Local Health Authority, Palermo, Italy; 3 Sicilian Health Department, Public Health and Environmental Risks Service, Palermo, Italy; 4 Department of Medicine and Surgery, “Kore” University of Enna, Enna, Italy; 5 Geriatric Unit, Department of Internal Medicine and Geriatrics, University of Palermo, Via del Vespro, 141, Palermo, 90127 Italy

**Keywords:** Vaccination, Frailty, Older people, Anti-pneumococcus, Influenza

## Abstract

Vaccination, particularly against pneumococcus and influenza, is a low-cost primary prevention, useful to avoid hard complications, particularly among frail older people. In this pilot study, we aimed to assess the effect of a strategy for influenza and pneumococcal vaccination for outpatients mainly affected by cognitive or endocrinological conditions, evaluating what could stimulate or demotivate vaccination among older people. This study was conducted during the 2023–2024 influenza season at the outpatient clinics in Palermo, Italy. A total 76 patients were included. More than half of the patients could be considered as pre-frail and about 20% frail, according to a comprehensive geriatric evaluation. Among patients, 46.05% received only vaccination against pneumococcus, 28.95% both vaccinations, and 25.0% only against influenza. Compared with the previous seasons, a 19.5% increase of influenza and 90.2% of pneumococcal vaccine uptake was observed. Side effects of vaccination were the main reason of the previous rejection, namely 76.9% for influenza and 53.8% for anti-pneumococcal vaccination. In conclusion, our study indicates how a new vaccination strategy in different settings could be feasible. Proposing influenza and pneumococcal vaccination for frail older outpatients could be an effective instrument to improve immunization coverage that is still low among older people.

**Supplementary Information:**

The online version contains supplementary material available at 10.1007/s40520-024-02824-5.

## Introduction

The influenza epidemic is a relevant public health challenge affecting thousands of people every year. According to the Italian Ministry of Health, this lead to an important budget increase caused by the management of this pathology, its complications and the rise of control procedures [1]. The European Center for Disease Control (ECDC) showed how in Europe approximately 70,000 people died for reasons strictly connected with influenza every year [2]. Pneumonia linked to influenza is a major cause of death in Italy, especially affecting older frail, and immunocompromised adults, and is listed among the top ten causes of mortality in our country [3].

Vaccination is certainly the best strategy to avoid influenza complications and there is agreement about its recommendation for frail people [4, 5]. The World Health Organization (WHO) guidelines state how a good control of influenza virus and transmission among the general population is a minimum of 75% vaccination coverage for influenza. Among frail people, coverage should be 95% in order to avoid probable negative consequences [1, 6]. In Italy, there are extensive vaccination advices to the population and vaccination is administered at no cost. However, the vaccination rate is still low: recent estimates reported that, also in older people, the influenza vaccination rate is just above 50% [7]. A cross-sectional computer-assisted web interviewing survey of 10,001 Italian citizens, conducted in 2023, underscored the negative impact of a lack of awareness that a person is in a priority group for influenza vaccination and the profound influence of social circles on vaccination decisions [8].

The available microbiological, epidemiological, and modeled data indicate that there is a substantial burden of disease attributable to *Streptococcus Pneumoniae* in adults ≥ 50 years of age [9]. These infections could cause community-acquired pneumonia and invasive pneumococcal disease, such as sepsis and acute meningitis. In the frail population, usually affected by several comorbidities, the risk of severe and lethal infections, is particularly high [10].

Increasing evidence shows that vaccination against influenza and pneumococcus based only on traditional primary care may not guarantee high vaccination adherence among frail people [11]. The main cause of failure could be the lack of a right vaccine delivery system to provide vaccines properly to persons in need, or even an improper use of vaccines, vaccine ineffectiveness at the time of use, and factors related to patients’ attitudes and knowledge [12]. The real power of vaccine catch-up instruments in rising vaccination coverage has been proven and several policies could be approved, such as phone calls with a vaccination remind, endorse immunization through informative interventions or attempts to vaccine risk groups in different settings from the primary care [13].

The Italian Ministry of Health, in addition to the usual national vaccination campaigns against influenza and pneumococcus, requests each regional administration to improve health strategies for population vaccination coverage [1]. In particular, at the beginning of the 2022–2023 influenza season, the Health Authority of Sicily produced a decree asking all hospitals, community dwelling and health care facilities to vaccinate against influenza virus all patients who were part of at-risk care groups before discharge [14].

Given this background, we aimed to assess the effect of a new plan for influenza and pneumococcal vaccination coverage dedicated to frail older outpatients, mainly affected by cognitive and endocrinological disorders, and to evaluate what could stimulate or demotivate influenza or pneumococcal vaccine uptake among older outpatients attending our ambulatories for different reasons, such as the evaluation of cognitive profile. This could represent a new strategy in the worldwide panorama of vaccination plans, attempting to reach as many persons as possible.

## Materials and methods

### Patients and procedures

A cross-sectional study took place from December 1, 2023, to February 29, 2024, in the Geriatrics section at the University Hospital “Policlinico P. Giaccone” in Palermo, Italy. Participants were patients attending outpatient clinics focused on managing cognitive disorders and endocrinological conditions such as diabetes and osteoporosis. During this period, these clinics saw 300 patients. Free vaccinations were offered to everyone over 60 years old in people without previous vaccination against pneumococcus.

The 2023-24 influenza vaccination campaign was carried out in these clinics, utilizing brochures, posters, and direct communication to inform about the risks and complications of influenza infection and the benefits of vaccination, especially for vulnerable individuals. A team of physicians from the Geriatrics Section of the Department of Health Promotion, Mother and Childcare, Internal Medicine, and Medical Specialties at the University of Palermo provided vaccinations every weekday while the clinics were open, from Monday to Friday. Collaboration with the medical and nursing staff helped identify eligible candidates for vaccination.

The Ethical Committee Palermo 1 approved the study at a meeting on December 12, 2023 (protocol no. 07/2023).

### Technical characteristics of the vaccinations proposed

Sicily region for the season 2023-24 indicated that all the physicians belonging to public health care structures may directly vaccinate the patients for influenza and for pneumococcus, whilst other vaccinations (e.g., COVID-19) are not still permitted in all settings (17). Vaccines were formulated according to Food and Drug Administration recommendations. Since High-Dose (HD) Influenza Vaccine is dedicated to frail patients typical of our outpatient clinics, we only administered this kind of vaccination. IIV4-HD (Efluelda^®^ฏ, Sanofi Pasteur) contained 60 µg of HA per strain. This vaccine is produced in embryonated chicken eggs, inactivated with formaldehyde, and split with a nonionic detergent.

Similarly, we used a 20‑valent pneumococcal conjugate vaccine (PCV20; Prevnar 20^®^; Apexxnar^®^), developed by Pfizer for active immunization for the prevention of pneumococcal infections. PCV20 has a similar structure and formulation to 13-valent PCV with the addition of seven capsular polysaccharides to target seven further *S. pneumoniae* serotypes (8, 10 A, 11 A, 12 F, 15B, 22 F and 33 F) associated with invasive pneumococcal disease with high mortality rates and antibiotic resistance. PCV20 has been approved for active immunization for the prevention of pneumonia and invasive disease caused by *S. pneumoniae* in adults since June 2021 in the USA and since February 2022 in the EU.

Both vaccines were provided in ready-to-use 0.5-ml syringes and administered intramuscularly, in the deltoid muscle site.

### Questionnaires

A validated and structured questionnaire, already used in other experiences among in hospital patients, was administered to all eligible patient for vaccination [15]. The questionnaire was addressed to the patients with the aim of identify factors associated with influenza and pneumococcus vaccine uptake. According to previous literature, the following items were investigated: personal and socio-demographic data such as, age, gender (male or female), education ( no schooling, primary school, middle school, high school, university degree), marital status (married, divorced, single, widow) health status (flu in the last five years, and if yes in which year), and behavior such as smoking (no smoker, former smoker, smoker), previous influenza, COVID-19 or *Pneumococcus* vaccination in the last five years [16, 17]. We specifically investigated if vaccinations were suggested or not by general practitioners (GP), and the reasons of not being vaccinated in the past (fear adverse effects, ineffective vaccine, others).

Moreover, we collected information about the presence of multidimensional frailty using the brief version of the selfie Multidimensional Prognostic Index (MPI) [18]. Brief-SELFY-MPI is a prognostic tool that demonstrates strong agreement with the standard version of the MPI. Supplementary Table 1 shows the eight domains of Brief-SELFY-MPI. Brief-SELFY-MPI maintains the multidimensional value of the full version by including eight domains. Each domain is assigned a risk rating: low risk = 0, moderate risk = 0.5, and high risk = 1 [18]. A score less than 0.33 indicates robust patients, between 0.33 and 0.66 pre-frail, and over 0.66 frail [18].

For patients with cognitive impairment, the questionnaire was completed by their relatives, if needed

### Outcomes

The main outcome of our pilot study was to assess the effect of a new plan for influenza and pneumococcal vaccination coverage dedicated to frail outpatients, mainly affected by cognitive and endocrinological disorders, and to evaluate what could stimulate or demotivate influenza or pneumococcal vaccine uptake among outpatients attending our ambulatories. Objectively, we measured these outcomes using self-reported information permitting the help of the caregiver, if requested.

### Sampling

During this period, our ambulatories evaluated 300 patients. Free vaccinations were offered to everyone over 60 years old. Being a pilot study, we did not calculate any sample size cut-off, but we consecutively included all outpatients interested in participating in the study, using a convenience sampling.

### Statistical analysis

All collected data were analyzed using SPSS 26.0 statistical software. The normality of the distribution for the quantitative variables was assessed with the Skewness and Kurtosis test. Mean and standard deviation (SD) was chosen for normal distribution of these variables, while median and interquartile range (IQR) was used for non-normal distribution. The absolute and relative frequencies were calculated for the qualitative variables. Study participants, according to gender, were compared using Chi-squared or Fisher exact tests, for categorical variables and t-test, for continuous variables.

## Results

**Fig. 1:**
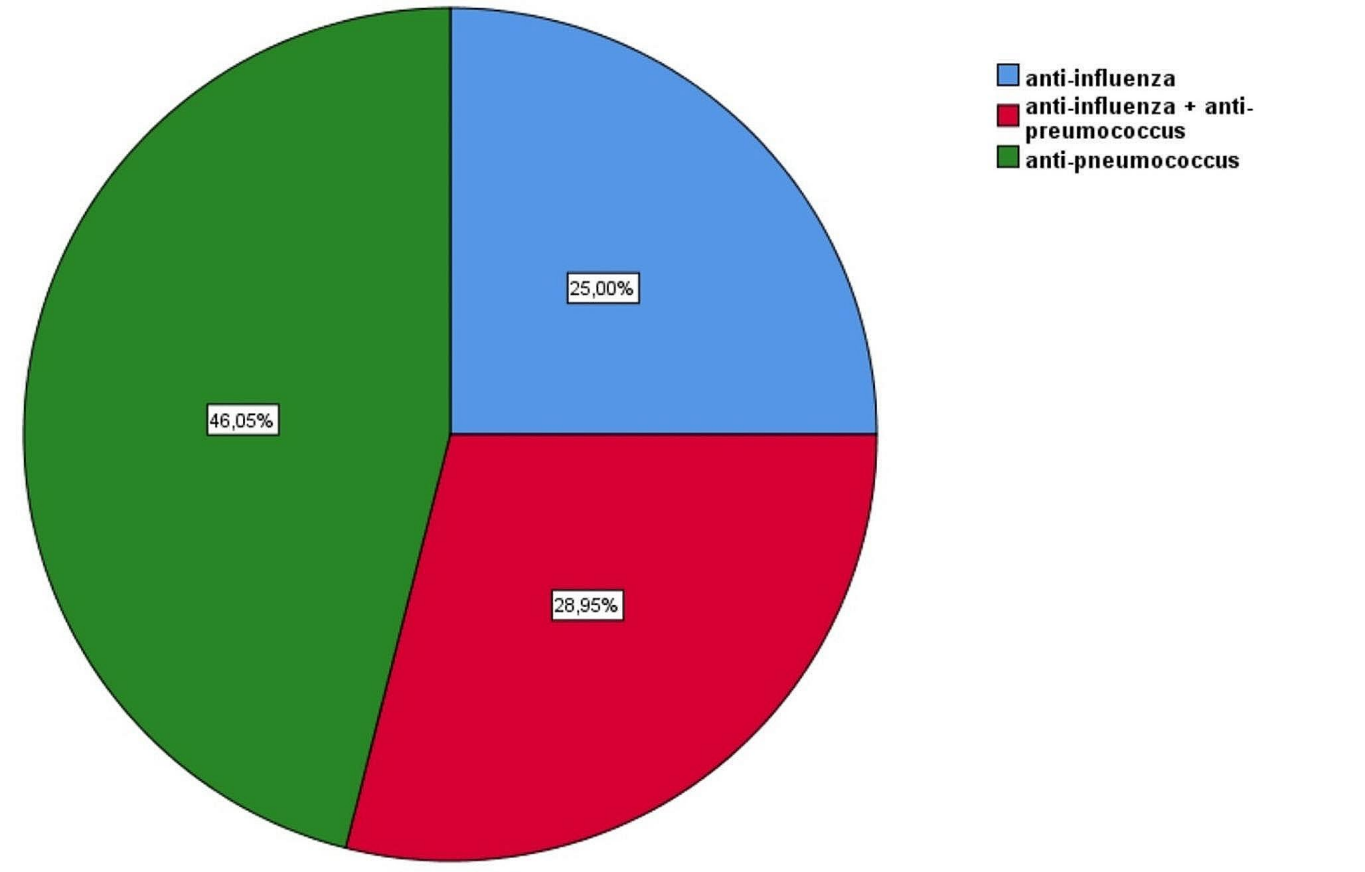
Prevalence of vaccinations done during season 2023-24

Overall, 300 patients were evaluated, a total of 76 outpatients during the seasons 2023-24, were included, 194 reported they were already vaccinated against influenza or Pneumococcus and 30 refused vaccination. Figure 1 shows the vaccinations carried out during the season 2023-24: 46.05% received only vaccination against pneumococcus, 28.95% received the combination of anti-pneumococcus and influenza and 25.00% only influenza.

.

Table 1 shows the most important descriptive characteristics of the population included. Overall, the 76 patients aged a mean of 76.1 years (SD = 7.5), with a range between 60 and 96 years. The majority of participants aged between 70 and 80 years (47.4%), followed by 80–90 years (26.3%);5.3% aged more than 90 years. Participants were prevalently females (69.7%). The large majority of the patients accessed to the outpatient clinic for cognitive disorders (71.1%), followed by endocrinology outpatient clinic (diabetes and osteoporosis) (29.9%). More than half of the patients could be considered as pre-frail according to the Brief-SELFY-MPI, about 20% frail and only 25.0% robust. Over half of participants were married (53.9%),38.2% widow, and 2.6% single. Regarding educational level, 47.4% had elementary school license and 7.9% did not ever attend school, and only 6.6% reached a degree (Table 1). Finally, half of the patients never smoked, 30.3% were former smokers, and1 0.5% were actual smokers. No significant differences emerged by gender.

**Table 1:** Descriptive characteristics of a population of outpatients vaccinated for influenza and/or pneumococcus

Variable	Values	Mean values or frequency (% or SD)	Females (*n* = 53)	Males (*n* = 23)	*p*-value
Age	Mean age (SD)	76.1 (7.5) (range: 60–96)	76.1 (8.0)	76.1 (6.3)	0.98
	60–70 years	21.1	20.8	21.7	1.00
	70–80 years	47.4	47.2	47.8	
	80–90 years	26.3	26.4	26.1	
	> 90 years	5.3	5.7	4.3	
Frailty status	Robust	19 (25.0)	30.8	13.0	0.23
	Pre-frailty	41 (53.9)	51.9	60.9	
	Frailty	16 (19.7)	17.3	26.1	
Ambulatory	Cognitive disorders	54 (71.1)	69.8	73.9	0.22
	Endocrinology	22 (29.9)	30.2	26.1	
Civil status	Married	41 (53.9)	50.9	60.9	0.41
	Widow	29 (38.2)	37.7	39.1	
	Single	2 (2.6)	3.8	0	
Educational level	Elementary	36 (47.4)	47.2	47.8	0.95
	Medium school	20 (26.3)	28.3	21.7	
	High school	1 (1.3)	1.9	0	
	Degree	5 (6.6)	5.7	8.7	
	No scholarity	6 (7.9)	7.5	8.7	
Smoking status	Actual	8 (10.5)	11.3	8.7	1.00
	Former	23 (30.3)	26.4	39.1	0.14

Table 2 shows the most important information about vaccinations received. Overall, 53.9% of the participants interviewed reported that they had influenza-like symptoms during the five previous years to the vaccination received in 2023-24. The majority reported signs or symptoms typical of influenza for only one season, and29.3% reported that this symptomatology was present for both seasons. When we asked for the role of GP in improving vaccination coverage, 7.9% of the interviewed participants reported that GPs did not indicate to get vaccination against influenza; similarly, half of the GPs did not suggest to vaccinate against pneumococcus.

**Table 2:** Characteristics about vaccinations for influenza and pneumococcus of a population of frail older outpatients

Variable	Values	Influenza (*n*,%)	Pneumococcus (*n*,%)
Influenza-like symptoms last 5 years	Yes	41 (53.9)	/
Number of seasons with influenza-like symptoms	1	19(46.3)	/
	2	12 (29.3)	
	3	5 (12.2)	
	4	5 (12.2)	
Suggested by GP	Yes	64 (84.2)	11 (14.5)
	No	6 (7.9)	38 (50.0)
	Don’t know/remember	6 (7.9)	27 (35.5)
Vaccination in the past	Yes	59 (77.6)	6 (7.9)
	No	15 (19.7)	68 (89.5)
	Don’t know/remember	2 (2.6)	2 (2.6)
If no, what’s the reason	Not suggested by GP	1 (8.3)	3 (23.1)
	Felt not efficacious	1 (8.3)	1 (7.7)
	Fear of adverse events	10 (76.9)	7 (53.8)
	Other reasons	/	2(15.4)

Finally, we also asked about vaccinations received in the past 5 years. It is noteworthy that 19.7% of the patients did not get vaccination against influenza, as well as 89.5% against pneumococcus, showing how our new strategy for vaccination coverage in these patients had good results. When we investigated the main reason for not getting vaccinations, the patients referred in 8.3% of the cases that the vaccination against influenza was not indicated by their GP, 8.3% did not feel that the vaccination against influenza could be efficacious, and 76.9% were worried about side effects. Similarly, when we asked for the same information for pneumococcus, the patients answered in 23.1% of the cases that this vaccination was not indicated by their GPs and more than half of the participants (53.8%) were worried about possible side effects (Table 2).

## Discussion

The first aim of this study was to assess the effect of a new plan for vaccination coverage in ambulatories dedicated to frail older accessing for other reasons, e.g., the evaluation of cognitive status or bone health or diabetes. Moreover, we evaluated what could stimulate or demotivate older people for influenza vaccine uptake in an age group commonly considered at higher risk and very vulnerable to infections. As far as we know, this is the first experience considering frail outpatients’ vaccination.

In our study we reached, for the first time, a very old population, since most participants aged between 70 and 80 years, followed by 80–90 years. Our study proposed a new concept of proactive vaccination: this strategy may be applied in territories where there is a shortage of GPs or where the Departments/Districts are not able to meet the needs of older population. Finally, regarding the period in which to vaccinate the older population, a key article strongly emphasizes the need to overcome the old concept of seasonal vaccination with a more active intervention, such as that shown in our work [19]. Nowadays, vaccination throughout the year is essential, not concentrating the wide choice of available vaccines only in the winter season.

The administration of vaccines in hospital settings is a plan encouraged by the WHO to decrease “wasted occasions” in older adult vaccination, increasing the distribution of health services and endorsing a real interaction among healthcare professionals [20, 21].

To the best of our knowledge, our experience is one of the few made in an outpatient setting, used as an instrument to develop vaccination coverage in older populations. Previous experience conducted in hospital settings include an Italian study among 248 patients demonstrated that offering influenza vaccination to hospitalized patients could be an effective strategy to increase vaccination coverage [15], but vaccination was only for hospitalized patients, not effective enough to reach the entire susceptible population. There are few experiences of influenza vaccination among outpatients, such as a South-American study of 465 participants with a median age of 37 years old [22] and without a real definition of frail patients according to geriatrics standards; or an American study of 7182 participants, where vaccines were administered only in specific medical reasons such as in allergy, infectious disease, pulmonary, and rheumatology ambulatories, with good results in term of vaccination rates [23]. However, participants were part of different departments, with great difficulties in communicating and managing different professionals, while in our case, different specialists of the various outpatient clinics belonged to the same department. Our work significantly differs from these important experiences since it is remarkable that more than 70% of the vaccinated participants in our ambulatories could be considered as pre-frail or frail from a multidimensional point of view.

People with dementia are at a greater risk of complications from respiratory infections [24]; therefore vaccinations against influenza and pneumococcal disease can benefit individuals with dementia by reducing both mortality and morbidity [25]. The large majority of the participants in our study accessed the outpatient clinic for cognitive disorders. A recent systematic review and meta-analysis showed that influenza vaccination was associated with a significantly lower risk of dementia, suggesting the role of this vaccination in the prevention of this geriatric syndrome [26].

Regarding pneumococcus, growing evidence suggests that pneumococcal vaccine reduces pneumonia and lower respiratory tract infections more broadly, including protecting against viral-associated respiratory diseases. In our study 28.95% received the co-administration of anti-pneumococcal and influenza vaccination and this could have possible additive effects: a meta-analysis showed how this association was associated with a significantly lower pneumonia rate than influenza vaccination alone, and with a significantly lower all-cause mortality rate than influenza vaccination alone [27], supporting the role of concomitant anti-pneumococcal and influenza vaccination for older people.

Another important finding of our work is that, with our experience, we increased by 20% the vaccination against influenza, as well as 90.2% against pneumococcus. This kind of patients would probably never be included in vaccination campaign, because of their frailty condition. In our setting we administered an high-dose and adjuvanted inactivated influenza vaccination, specifically developed to provide enhanced immune responses in older adults [28], who generally have low responses mainly due to immunosenescence, comorbidities and frailty.

Our study analyzed vaccination coverage in the past 5 years, and a sadly higher percentage of older patients did not get vaccination against influenza or against pneumococcus. Although vaccine hesitancy has existed among a small percentage of people for centuries, its harmful effects are likely to be more pronounced during the COVID-19 pandemic than ever before [29]. The reasons for hesitancy in vaccination are usually complex and include socio-demographic, physical, and psychosocial factors [30].

Investigating the main reasons for not getting vaccinated about influenza and pneumococcus, more than 70% reported that they were worried about side effects for the first one, and almost half of the patients for the second one, showing how these patients should be advised in their choice of vaccination. At the same time, another possible reason of the hesitancy is that health care professionals may find many problems in the communication with older people about the importance of vaccination [31].

The present results were very encouraging for us and testify trust between outpatients and professional in our outpatient clinics, which is a crucial aspect in order to lead to reasonable goals. We observed that numerous patients listened to our reasons to get vaccinated, sometimes more than to the GP, with whom they may not have had a valid relationship, confiding in us that given their fragile condition, they would not go to the GP, highlighting the great impact of this new strategy of vaccination. Our results reported that in 8.3% of the cases, the vaccination against influenza, and in 23.1% of the cases against pneumococcal, were not indicated by the GP. Vaccine hesitancy among GPs could explain these data: a French study of 1712 randomly selected GPs, showed that 16–43% of them, sometimes or never recommended at least one specific vaccine to their target patients [32].

The findings of our study must be interpreted within its strengths and weaknesses. Firstly, this study is the first experience, to the best of our knowledge, of frail outpatients vaccination, representing a unique experience. Secondly, proposing influenza and pneumococcal vaccination for outpatients could be an effective instrument to improve immunization coverage. Third, opening vaccination coverage to geriatricians could extend the range of specialists engaged in vaccination, with higher coverage among frail patients. The main limitation is the lack of a control group: therefore, no comparison was made between a group receiving vaccinations and a group not receiving it or using before-and-after study. However, our study is a pilot experience and we did not a priori plan to have a control group. In this sense, we are not able to explore which factors could be associated with a lower or higher vaccination rate among older frail outpatients. Additionally, information about vaccination and signs or symptoms typical of influenza were self-reported, which may not exclude the possibility of bias. Thirdly, the small population included may not be representative of all frail older people designed for vaccine coverage in the general population, even if our data suggest that the population targeted was frail from different points of view, including comorbidities, polypharmacy, and disability.

## Conclusions

Our study demonstrated how a new vaccination strategy in the outpatient clinic setting is possible, especially for frail people. This is the first experience, to the best of our knowledge, of frail outpatients vaccination, representing a unique experience. Proposing influenza and pneumococcal vaccination for outpatients could be an effective instrument to improve immunization coverage. If our new concept of vaccination would beapplied in a large scale in every hospital or health care facility, a significant improvement in influenza and pneumococcal vaccines rates for frail people could be reached. However, to validate our preliminary experience, it is necessary to include more subjects, a more diverse population, and criteria for measuring their effectiveness.

## Electronic supplementary material

Below is the link to the electronic supplementary material.

Supplementary Material 1

## Data Availability

Data will be available after to motivated request to corresponding author.
