# Supplementary material for: Management of the vaccination campaign in a population of frail older outpatients affected by cognitive or endocrinological conditions: a pilot study in Italy
Source: Aging Clin Exp Res. 2024 Aug 30;36(1):179. doi: 10.1007/s40520-024-02824-5 (PMC11364585; doi:10.1007/s40520-024-02824-5)
Supplement: Supplementary file 1 — Supplementary Material 1 [file 40520_2024_2824_MOESM1_ESM.docx]

**Supplementary table 1T** The eight domains of Brief-MPI

-Activities of Daily Living (ADL): Derived from the activities of daily living assessment.

-Instrumental Activities of Daily Living (IADL): Based on the instrumental ADL assessment.

-Cognitive Assessment: Using the Short Portable Mental Status Questionnaire (SPMSQ).

-Mobility Assessment: Evaluated using the Barthel Mobility Index.

-Nutritional Assessment: Conducted with the Mini-Nutritional Assessment Short Form.

-Comorbidities: Evaluated using the Cumulative Illness Rating Scale (CIRS).

-Number of Medications in Use.

-Cohabitation Status.

The first seven domains are assessed with dichotomous responses (yes/no or right/wrong). The cohabitation status is scored as 0 for individuals living with family, 0.5 for those institutionalized, and 1 for those living alone.
